# Risking Your Life without a Second Thought: Intuitive Decision-Making and Extreme Altruism

**DOI:** 10.1371/journal.pone.0109687

**Published:** 2014-10-15

**Authors:** David G. Rand, Ziv G. Epstein

**Affiliations:** 1 Department of Psychology, Yale University, New Haven, CT, United States of America; 2 Department of Economics, Yale University, New Haven, CT, United States of America; 3 School of Management, Yale University, New Haven, CT, United States of America; 4 Pomona College, Claremont, CA, United States of America; Centre national de la recherche scientifique, France

## Abstract

When faced with the chance to help someone in mortal danger, what is our first response? Do we leap into action, only later considering the risks to ourselves? Or must instinctive self-preservation be overcome by will-power in order to act? We investigate this question by examining the testimony of Carnegie Hero Medal Recipients (CHMRs), extreme altruists who risked their lives to save others. We collected published interviews with CHMRs where they described their decisions to help. We then had participants rate the intuitiveness versus deliberativeness of the decision-making process described in each CHMR statement. The statements were judged to be overwhelmingly dominated by intuition; to be significantly more intuitive than a set of control statements describing deliberative decision-making; and to not differ significantly from a set of intuitive control statements. This remained true when restricting to scenarios in which the CHMRs had sufficient time to reflect before acting if they had so chosen. Text-analysis software found similar results. These findings suggest that high-stakes extreme altruism may be largely motivated by automatic, intuitive processes.

## Introduction

Cooperation, defined as paying a cost to give a greater benefit to one or more others, is an integral part of human behavior and a cornerstone of human societies [1]–[12]. While cooperative behavior improves group welfare, the personal incentive to be selfish poses a challenge to cooperation. A large literature across numerous fields has sought to understand the origins of cooperative behavior, and numerous mechanisms for the evolution of cooperation have been identified [5], [8]. These include direct reciprocity [13]–[22], indirect reciprocity [23]–[32], population structure [22], [33]–[39], group selection [40]–[46], and kin selection [47], [48]. In addition to these ultimate explanations for cooperative behavior, it is also of both scientific and practical importance to understand the proximate *psychological* underpinnings of cooperation [49]–[54].

A growing literature explores cooperation, and prosocial behavior more generally, using a dual process framework, in which decisions are conceptualized as resulting from the competition between two cognitive systems: one that is fast, automatic, intuitive, and often emotional, and another that is slow, controlled, and deliberative [55]–[61]. We follow conventions in evolutionary biology and define prosocial behaviors as those which benefit others; altruistic behaviors as prosocial behaviors which are individually costly; and cooperative behaviors as altruistic behaviors where the cost paid is smaller than the benefit provided to others (i.e. cooperation is costly and non-zero sum).

A range of recent laboratory studies have examined the role of intuition and deliberation in cooperation and altruism using economic games. In these games, players make choices which affect the amount of money they and others earn. For example, a canonical game for studying cooperation is the Public Goods Game, where a group of participants simultaneously choose how much money to keep for themselves versus how much to contribute for the benefit of the other group members; and for altruism is the Dictator Game, in which one participant unilaterally chooses how to divide a sum of money with another person. Experiments have manipulated cognitive processing while participants played these games, increasing the role of intuition by applying time pressure [62]–[65] and conceptual priming of intuition [63] to the Public Goods Game, and cognitive load [66]–[68], immediate rather than delay timing of payments [69], [70], and disruption of the right lateral prefrontal cortex [71] to the Dictator Game, and finding increases in participants' willingness to pay money to benefit others (although some other studies find null effects for some of these manipulations [72]–[74]). Furthermore, participants seem to project a cooperative frame onto neutrally framed Prisoner's Dilemma games [75], and analyzing free-text narrative descriptions of participants' decision processes during Public Goods Games finds that inhibition is associated with reduced cooperation, while positive emotion is associated with increased cooperation [76], [77].

The “Social Heuristics Hypothesis” (SHH) has been proposed as a theoretical framework to explain these results and predict potential moderators [62]. The SHH adds an explicitly dual process perspective to work on cultural differences [6], [78]–[81], norm internalization [82]–[85] and exchange heuristics [86], [87] in order to understand how intuition and deliberation interact to produce selfish or generous behaviors. The SHH postulates that we internalize strategies that are typically advantageous in our daily social interactions as intuitive default responses. When confronted with more atypical social situations, our automatic response is to continue to apply these daily life defaults; but then more reflective, deliberative processes can override these automatic defaults and shift our behavior towards that which is most advantageous in the specific context at hand. In sum, strategies which are advantageous (i.e. payoff-maximizing) in daily life interactions become automatized as intuitions, and are then over-generalized to less typical settings. Direct evidence for such spillovers comes from experiments where exposure to long or short repeated games influences subsequent behavior in one-shot anonymous interactions [85].

These laboratory experiments using economic games provide valuable insight into the cognitive underpinnings of cooperation and altruism: they offer a high level of control and precision, and make quantification easy. Although these games are very simple and decontextualized, there is evidence that game play is reflective of underlying moral values, and predictive of actual helping behavior in a task which is not obviously part of an experiment [88]. The question remains, however, of how intuition and deliberation function outside the laboratory, particularly in contexts where helping others is more costly than it is in these low stakes games. One piece of recent evidence in this vein comes from a correlational study showing that individuals with little self-control are more likely to make sacrifices for the benefit of their romantic partners [89]. Classic work studying more contextualized helping behavior, such as agreeing to help another student study [90] or taking electric shocks on behalf of another participant [91] has suggested an important motivational role of empathy, implicating emotional (i.e. intuitive) processes. Finally, a recent study examined the extremely costly behavior of kidney donation (albeit not from a dual process perspective) and found that across the United States, kidney donation was more likely in areas with higher subjective well-being [92].

In the present paper, we explore the role of intuition and deliberation in the highest cost of all decisions: risking one's life to save a stranger. It is obviously infeasible and unethical to study actual behavior of this kind in the laboratory, and while surveys of hypothetical extreme altruism can be very informative (e.g. [93]), they are inherently limited, as most participants have no experience with such situations and there is reason to doubt the accuracy of self-reports in this domain.

Instead, we examine actual acts of extreme altruism using archival data: published interviews with people awarded medals by the Carnegie Hero Fund Commission for risking their lives to an extraordinary degree saving or attempting to save the lives of others. Although we refer to this behavior as extreme altruism, we note that in most cases this behavior actually meets the definition of cooperation given above: when you risk your life to save another person, the aggregate outcome is better than if you chose not to (as long as you have a good enough chance of saving the other person and not dying in the process).

Based on the evidence of intuitive cooperation from low-stakes economic games, and the role of emotion in more contextualized helping, we predicted that the interviews with these Carnegie Hero Medal Recipients (CHMRs) would reveal that their heroic acts were motivated largely by automatic, intuitive responses. In two studies, we confirm this prediction. In Study 1, we had participants read excerpts from the CHMRs' interviews in which that described their decision-making process, and rate them as relatively intuitive versus deliberative. In Study 2, we analyzed the level of inhibitory language in these excerpts using a computer algorithm.

## Study 1

### Methods

#### Extreme altruist stimuli

To collect the CHMR statements, we used the Carnegie Hero Fund Commission website to compile a list of all CHMRs between Dec 17 1998 and Jun 27 2012. To qualify as a CHMR, a person must be a civilian who voluntarily risks his or her life to an extraordinary degree while saving or attempting to save the life of another person; the rescuer must not be responsible for the safety of the victim; and the event must occur in the United States or Canada.

We then cross-referenced this registry with local, regional and national online news sources, and collected any interviews with the CHMRs discussing their heroic action. We extracted all quoted material spoken directly by the CHMR in which they described the decision-making process involved in their altruistic activity (i.e. *why* they did what they did). We removed as much material indicating *what* specific action they had taken as possible, without harming the intelligibility of the statements. Below we include the results of a pilot study which used the totally unedited CHMR quotes and found very similar results to the edited texts.

In total we collected 51 statements in which CHMRs described their decision-making (see Material S1 for each CHMR statement). The average CHMR age was 36.4 years (min 15, max 77), and 82% of the CHMRs were male. In terms of geographical location, the CHMRs were overwhelmingly American (2 out of 51 were from Canada), with 20% of the Americans coming from states in the West, 20% from the Mid-West, 29% from the South, and 31% from the Northeast.

To give some sense of the CHMRs and their statements, here we reproduce several examples. Christine Marty, a 21 year college student, rescued a drowning 69-year-old trapped in a car during a flashflood, and stated “I'm thankful I was able to act and not think about it.” Daryl Starnes, a 70-year-old man, climbed into a burning vehicle to rescue a 48-year-old woman trapped inside after a car accident, and stated “I just did what I felt like I needed to do. You don't think about someone making that big a deal out of it.” Kermit Kubitz, a 60-year-old man, witnessed a man in a bakery stab a 15-year-old girl without provocation, and immediately engaged the man and was himself stabbed. He stated “I had only two thoughts: one, I have to get him out of the door, and two, oh my God, this guy could kill me, too. I ended up on my back with the knife in my ribs, I think it was just instinct. Kind of like my tendency, that nobody in my platoon is going to get attacked without me doing something, if it were my daughter, you'd do it for me. You'd do it in an instant. And I'd do it for you.”

#### Control stimuli

To create corpora of control statements for comparison to the CHMR statements, we used statements generated in a previous study where subjects were asked to write about a time in their life where either following their intuition or carefully reasoning through a problem led to a good outcome [63]. From these statements, we selected 25 describing the use of intuition and 25 describing the use of deliberation. In our selection of control statements, we attempted to choose statements that were similar in format and length to the CHMR statements for maximum comparability, and that most clearly reflected the indicated style of decision-making. The mean length of the CHMR statements was 48.8 words, of the intuition controls was 50.9 words, and of the deliberative controls was 58.0 words (no significant differences in length, p>0.05 for all pairwise t-tests, see Material S1 for each control statement).

#### Ratings of intuitiveness vs deliberativeness

To measure the extent of intuitive versus deliberative decision-making described in the statements, we had participants rate the statements using a 7-point scale (“Intuitive/Fast” to “Reasoned/Slow”). A total of 312 participants were recruited for Study 1 using Amazon Mechanical Turk [94], [95], based on a target of 100 subjects in each of the three conditions (CHMR statements, intuitive controls, deliberative controls; all data available in the Supplemental Material). Data was collected in a single run, and no additional subjects were recruited subsequently. Participants were paid $0.30 for completing the study. Each participant first read a set of instructions explaining the concepts of intuition and deliberation, and was shown sample statements that were highly intuitive and highly deliberative. Intuitive decisions were described to subjects using the terms fast, snap judgment, not involving much thought, automatic, emotional, and effortless. Deliberative decisions were described to subjects using the terms slow, carefully weighing options, involving a lot of thinking, controlled, rational, and effortful. Each participant then rated 16 randomly selected statements (by chance, 2 subjects were not shown any intuitive control statements, and another 2 subjects were not shown any deliberative control statements; these subjects are excluded from subsequent analysis).

#### Estimating the time CHMRs had to act

To address the possible concern that CHMRs must by definition act automatically, because extreme altruism often requires immediate action, an additional 106 participants were recruited using Mechanical Turk to assess the amount of time each CHMR had in which to act before it would have been too late to save the victim. Again sample size was based on a target of 100 subjects per condition, and data was collected in a single run. Participants were paid $0.30 for completing the study. Participants were presented with descriptions of the scenarios faced by CHMRs taken from the Carnegie Hero Medal Foundation website, and asked to estimate the number of seconds the CHMR had to save the potential victim(s). Each participant read and rated descriptions of 10 randomly selected scenarios.

#### Ethics statement

This study was approved by the Human Subjects Committee of the Yale University Human Research Protection Program, and written informed consent was received from all participants.

### Results

**Figure 1:**
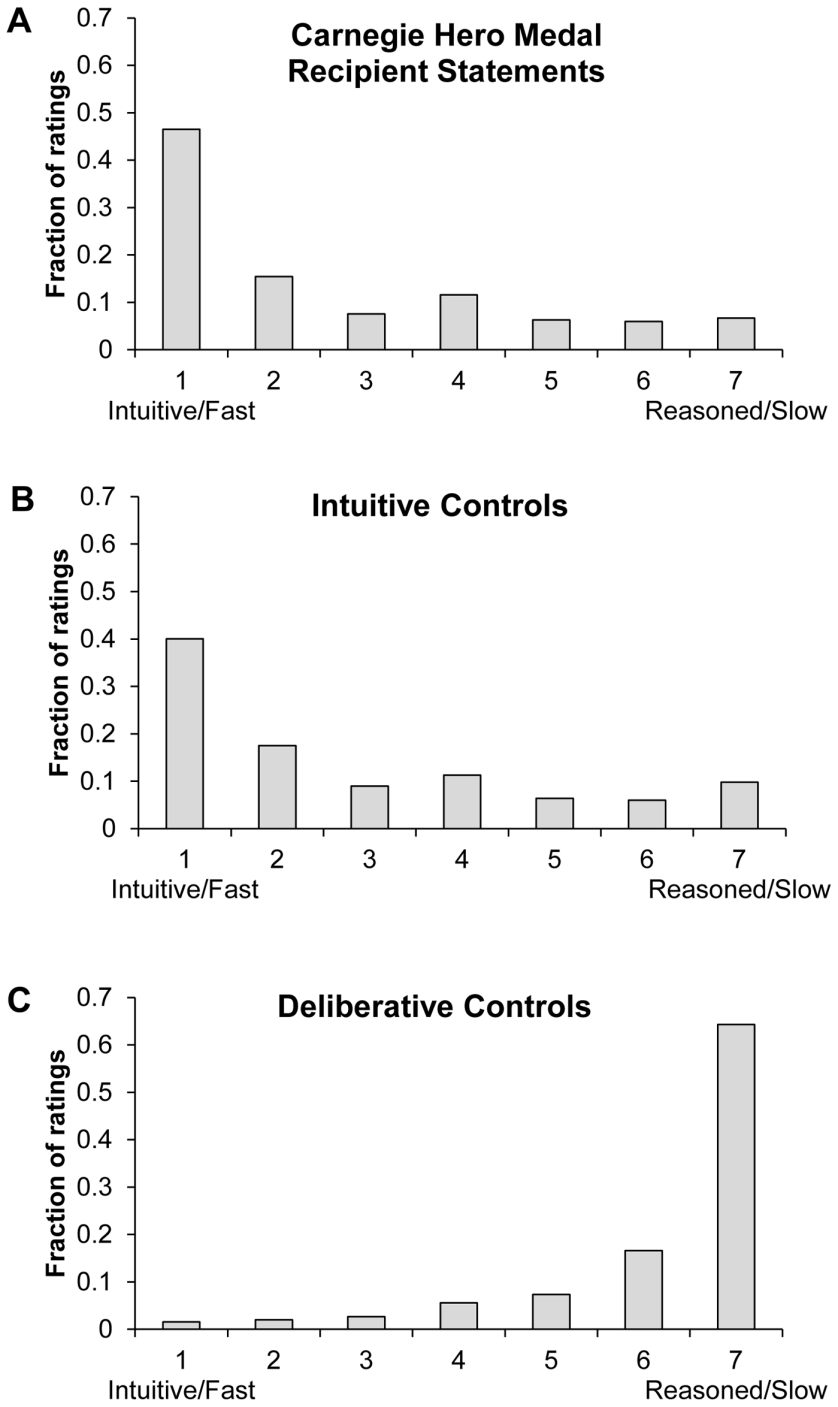
Distribution of ratings of CHMR statements (A), intuitive control statements (B) and deliberative control statements (C) in Study 2.

The intuitive versus deliberative ratings of the CHMR statements, the intuitive controls and the deliberative controls are shown in Figure 1.

As predicted, the CHMR ratings were strongly skewed toward “Intuitive/Fast.” The modal CHMR rating was the maximally intuitive value of 1 (46.5% of responses), and the mean rating was 2.61, which is significantly lower (i.e. more intuitive) than the scale mid-point of 4 (one-sample t-test, t(50) = −9.31, p<0.0001). Moreover, 92.2% of CHMR statements had a mean rating below the midpoint of 4. [Very similar results were found in a pilot study where 73 Mechanical Turk participants rated the full quotes from the CHMR interviews (rather than just the sections having to do with the decision-making process), as well as four additional CHMR statements which did not describe the decision-process at all and thus were omitted from our main analysis: the modal response was the maximally intuitive value (34.0% of responses); the mean rating was 3.18; and 80.0% of statements had a mean rating below 4.]

The results for the intuitive controls closely resembled those of the CHMR statements. The modal rating was also the maximally intuitive value of 1 (40.0% of responses), and the mean rating of 2.84 was significantly lower than the scale mid-point of 4 (one-sample t-test, t(24) = −7.44, p<0.0001). Moreover, 88.0% of intuitive control statements had a mean rating below the midpoint 4.

The results for the deliberative controls, however, looked starkly different. The modal response was the maximally *deliberative* value of 7 (64.3% of responses), and the mean rating of 6.23 was significantly higher (i.e. more deliberative) than the scale mid-point of 4 (one-sample t-test, t(24) = 22.4, p<0.0001). Moreover, 100% of deliberative control statements had a mean rating above 4.

Comparing the statement-average ratings across the three different types of statements, we find no significant difference between the CHMR statements and the intuitive controls (two-sample t-test, t(74) = −0.97, p = 0.33), while the deliberative controls were rated as significantly more deliberative than either the intuitive controls (two-sample t-test, t(48) = −18.3, p<0.0001) or the CHMR statements (two-sample t-test, t(74) = −16.1, p<0.0001). Qualitatively equivalent results are given by analysis at the level of the individual rating (one observation per subject per statement) using linear regression with robust standard errors clustered on subject, including indicator variables for intuitive and deliberative control conditions, and controlling for log10(statement length) and rater's age, gender and education level (intuitive control condition indicator, capturing the difference between CHMRs and intuitive controls, p>0.05; deliberative control condition indicator, capturing the difference between CHMRs and deliberative controls, p<0.001).

**Figure 2:**
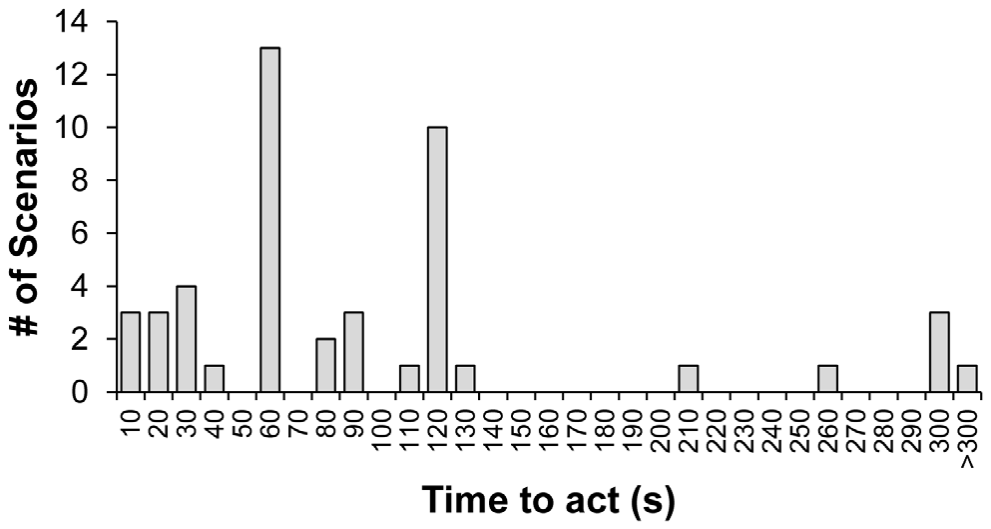
Median rating of number of seconds CHMRs had in which to act for each CHMR scenario.

We now ask whether these results hold when restricting our attention to scenarios it was not by definition necessary for the CHRM to act immediately in order to be effective. To do so, we calculate the median number of seconds participants estimated each CHMR had in which to act before it was too late. The distribution of median “times to act” for the 51 CHMR scenarios is shown in Figure 2. We see that in a substantial subset of the scenarios, the CHMRs did actually have a substantial amount of time to deliberate if they had chosen to do so. For example, in 71% the scenarios (36 out of 51), participants estimated the CHMR had at least 60 seconds before they had to act.

We continue to find that the CHMR statements are significantly more intuitive than the deliberative controls when restricting to scenarios where the CHMR had at least 60 seconds to act (t-test: t(59) = −16.3, p<0.0001), or at least 120 seconds to act (t-test: t(40) = −13.4, p<0.0001). Furthermore, we find no significant relationship between the number of seconds CHMRs had to act and ratings of the intuitiveness of their choice (linear regression: t = 0.83, p = 0.41; using log10-transformed times to act, t = 0.95, p = 0.35). Thus it does not seem that the intuitiveness of CHMR choices is the trivial result of them being in scenarios where automatic immediate responses were required.

Finally, we ask whether demographic characteristics of the CHMRs predict the extent to which their statements were rated as intuitive versus deliberative. We find no significant relationship between the rating of each CHMR's statement and their age, gender, or geographic region (ANOVA, p>0.05 for all), perhaps because of a relatively small sample size; although we note that the two Canadian CHMRs were rated as substantially more reflective (4.4) than the 49 Americans (2.53).

### Discussion

These results suggest that the decision-making processes described by the CHMRs were predominantly driven by intuitive, fast processing. While the pattern in these results is clear, there is a limitation of the design of Study 1: it is possible that our raters did not fully understand the constructs of intuition and deliberation that they were asked to use when rating the CHMR statements.

## Study 2

### Introduction

In Study 2, we address potential limitations stemming from Study 1's use of inexpert human raters by employing the Linguistic Inquiry Word Count (LIWC) software [96] to characterize the level of inhibition indicated in each statement. We predicted that CHMR statements would involve less inhibitory language than the deliberative controls, and would not differ from the intuitive controls.

### Method

Each of the CMHR statements, intuitive control statements, and deliberative control statements from Study 2 were analyzed using LIWC. The LIWC software analyzes the frequency of different types of words in a text, and rates the extent to which a range of social, cognitive, and emotional concepts are present in that piece of text. Given that the heart of most dual process theories involves deliberative responses exerting control to inhibit automatic responses, the LIWC category that maps most directly onto the dual process framework we employed in Study 1 is the ‘Inhibition’ category. To avoid issues related to multiple comparisons, we analyzed each statement's rating on only this one category, giving the statement a score of 0 if no inhibitory language was present (i.e. the LIWC Inhibition score was 0) and 1 otherwise. We used this binary classification rather than a continuous measure of number of inhibitory words because the distribution of word counts was extremely right skewed, making meaningful analysis difficult using a continuous measure.

### Results

A total of 13.5% of CHMR statements included inhibitory language. As predicted, inhibition was significantly less common among CHMR statements than deliberative controls, 40% of which contained inhibitory language (Pearson χ^2^(1) = 6.91, p = 0.009). Conversely, there was no significant difference in the prevalence of inhibitory language between the CHMR statements and the intuitive controls, 8.0% of which included inhibitory language (Pearson χ^2^(1) = 0.49, p = 0.48). Similar results are found using a logistic regression with robust standard errors predicting presence of inhibitory language, including indicator variables for intuitive and deliberative control conditions, and controlling for total word count (intuitive control condition indicator, capturing the difference between CHMR and intuitive controls, p>0.05; deliberative control condition indicator, capturing the difference between CHMR and deliberative controls, p = 0.015).

## General Discussion

In two studies, we provided evidence that when extreme altruists explain why they decided to help, the cognitive processes they describe are overwhelming intuitive, automatic and fast. These results are consistent with previous evidence from the laboratory using low-stakes economic games, and suggest that these earlier findings may generalize to higher stakes settings outside the lab. In addition, our results align with theoretical predictions of the Social Heuristics Hypothesis [62], which suggests that extreme altruism may be a result of internalizing (and subsequently overgeneralizing) successful behavioral strategies from lower-stakes settings where cooperation is typically advantageous: helping others is usually in one's long-term self-interest in the context of most daily-life interactions with friends, family members and co-workers. This leads to the development of helping as an automatic default, which then sometimes gets applied in atypical settings where helping is extreme costly, such as the CHMR scenarios.

Studying extreme altruism presents major challenges, as such behavior cannot be enacted in the lab, and hypothetical survey measures are likely to have little to do with actual behavior in these extreme settings. Thus we sought out statements from actual extreme altruists. Our archival methodology, however, has numerous limitations. There may be bias in which CHMRs chose to give interviews, and which interviews were released by the press. There may also be bias in how CHMRs actually remember the incidents they are describing due to the emotionally arousing content of such memories [97]. In addition, we operated under the assumption that the extent to which the CHMR statements rated as intuitive corresponded to the actual intuitiveness of the action itself, but there may well be a disconnect between how CHMR describe their thought processes and what their actual thought processes were at the time. Thus more work clearly is needed to fully understand the cognitive underpinnings of extreme altruism, including direct (rather than archival) interviews and neurobiological investigation. Nonetheless, we believe that our results provide important insight, and hope that our results will stimulate further research on this topic.

## Supporting Information

Materials S1 Key explaining the data file together with screenshots of the web survey. This includes the text of each of the CHMR and control statements.
(PDF)

Materials S2 Comma-separated-values file containing raw experimental data.
(CSV)
