# Supplementary material for: Risking Your Life without a Second Thought: Intuitive Decision-Making and Extreme Altruism
Source: PLoS One. 2014 Oct 15;9(10):e109687. doi: 10.1371/journal.pone.0109687 (PMC4198114; doi:10.1371/journal.pone.0109687)
Supplement: Materials S1 — Key explaining the data file together with screenshots of the web survey. This includes the text of each of the CHMR and control statements. (PDF) [file pone.0109687.s001.pdf]

<u>Materials S1</u>

This document contains the experimental instructions for Rand & Epstein 2014, as well as a listing of the questions that go along with each column in the data file. In addition to the columns which contain the subject ratings for each statement (as listed below), there is an additional column for each statement listing it's word length (using the naming convention Wcarn1, Wcarn2, …), as well as a column listing the average number of seconds subjects reported that hero would have had in which to act before it was too late (using the naming convention Tcarn1, Tcarn2, …)

INSTRUCTIONS

In this study, we will ask what you think of a series of short statements. These are statements made by real people in interviews, not like the nonsense paragraph you just transcribed.

In particular, we want to know whether the decision was made intuitively, or whether it was the result of careful reasoning.

So, you will be asked to rate how intuitive versus reasoned each statement is.

To help you understand what we are asking, here are some definitions of what we mean by intuitive versus reasoned.

Intuitive decisions are characterized as:
* Fast
* Snap judgments
* Not involving much thought
* Automatic
* Emotional
* Effortless

Reasoned decisions are characterized as:
* Slow
* Carefully weighing options
* Involving a lot of thinking
* Controlled
* Rational
* Effortful

Here is an example of a very intuitive statement:

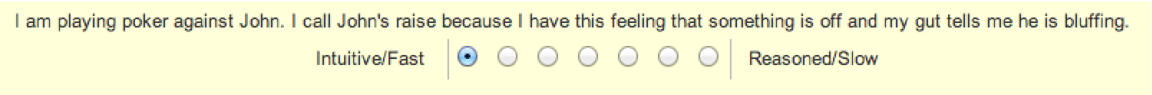

Here is an example of a very reasoned statement:

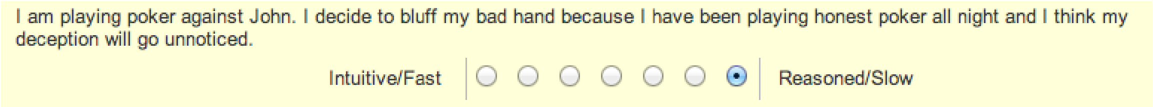
I am playing poker against John. I decide to bluff my bad hand because I have been playing honest poker all night and I think my deception will go unnoticed.

Intuitive/Fast ○ ○ ○ ○ ○ ○ ◉ Reasoned/Slow

Here is an example of a nuetral statement, i.e. one that has neither reasoned nor intuitive characteristics:

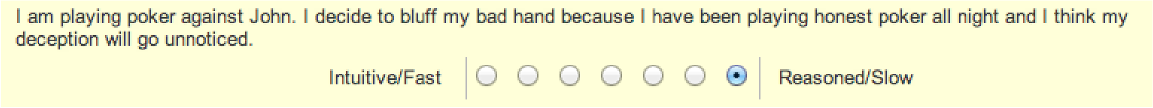
I am playing poker against John. I decide to bluff my bad hand because I have been playing honest poker all night and I think my deception will go unnoticed.

Intuitive/Fast ○ ○ ○ ○ ○ ○ ◉ Reasoned/Slow

When you are ready to continue to the questions, click the arrow below. You will then be taken to the first statement, and asked to rate it as intuitive versus reasoned.

<u>List of statements, in the format:</u>

Variable name Statement

carn1 I'm thankful I was able to act and not think about it.

	1 (1)	2 (2)	3 (3)	4 (4)	5 (5)	6 (6)	7 (7)
Intuitive/Fast:Reasoned/Slow (1)	❍	❍	❍	❍	❍	❍	❍

carn2 Honestly, in a situation like that, you're put in it and you just think he had to be stopped somehow. You don't think ... you just react. [I] just reacted; he just had to be stopped. When you think about what could have happened and think what didn't — you feel really blessed that Kirk was able to react that fast, Kirk was really the one who took the first step. I used him as inspiration and just followed my buddy.

	1 (1)	2 (2)	3 (3)	4 (4)	5 (5)	6 (6)	7 (7)
Intuitive/Fast:Reasoned/Slow (1)	❍	❍	❍	❍	❍	❍	❍

carn3 I didn't feel any pain at the time, I think adrenaline kicked in as my only thought was that I had to get to the women, I never felt any pain. The adrenaline just kicked in. I was trying to get there as fast as I could.

	1 (1)	2 (2)	3 (3)	4 (4)	5 (5)	6 (6)	7 (7)
Intuitive/Fast:Reasoned/Slow (1)	❍	❍	❍	❍	❍	❍	❍

carn4 When I was halfway across, I thought, this probably wasn't a good idea. When it happened, I had no doubt in my mind that I was going to do it.

	1 (1)	2 (2)	3 (3)	4 (4)	5 (5)	6 (6)	7 (7)
Intuitive/Fast:Reasoned/Slow (1)	❍	❍	❍	❍	❍	❍	❍

carn5 I just did what needed to be done. [A friend of my fathers] told me to go out there. I didn't think about it, I didn't have time to think about it. It just happened. There really wasn't anyone else there. It was instinct, I just think it was the adrenalin that pushed me to do that.

	1 (1)	2 (2)	3 (3)	4 (4)	5 (5)	6 (6)	7 (7)
Intuitive/Fast:Reasoned/Slow (1)	❍	❍	❍	❍	❍	❍	❍

carn6 The minute we realized there was a car on the tracks, and we heard the train whistle, there was really no time to think, to process it ... I just reacted. I think when we're forced into this kind of situation you become a different person. I never thought I'd be capable of doing something like that.

	1 (1)	2 (2)	3 (3)	4 (4)	5 (5)	6 (6)	7 (7)
Intuitive/Fast:Reasoned/Slow (1)	❍	❍	❍	❍	❍	❍	❍

carn7 I went ahead and just climbed through the fence and I don't remember ever feeling the electricity. . It was just here is the problem, here's what I need to do and something needed to happen. If nobody came to this woman's rescue, she would die. I didn't really take the time to think about what would happen.

	1 (1)	2 (2)	3 (3)	4 (4)	5 (5)	6 (6)	7 (7)
Intuitive/Fast:Reasoned/Slow (1)	❍	❍	❍	❍	❍	❍	❍

carn8 I can't say I really thought about my own life at the time. Just try to help, I mean I did what any normal person would do I mean you know I just kept thinking these were somebodies kids you know what I mean at the time my daughter was like 16 and I'm saying to myself you know if something god forbid would ever happen to her that I would hope someone would be there to help.

	1 (1)	2 (2)	3 (3)	4 (4)	5 (5)	6 (6)	7 (7)
Intuitive/Fast:Reasoned/Slow (1)	❍	❍	❍	❍	❍	❍	❍

carn9 For some strange reason a voice out of nowhere said don't worry about your own. He was a stranger a total stranger but you know what the mission was come completed I was chose for that. I felt chosen. I felt like I was the chosen one. Well you know what maybe 20 years ago I was supposed to be at a certain point. This thing that happened you know I had a gun pulled to my temple but you know it was a misfire so you know. I was almost dead. I was spared for a reason. I can do this. I can do this. When that voice said everything is going to be ok I knew everything was going to work out.

	1 (1)	2 (2)	3 (3)	4 (4)	5 (5)	6 (6)	7 (7)
Intuitive/Fast:Reasoned/Slow (1)	❍	❍	❍	❍	❍	❍	❍

carn10 I couldn't just stand there and do nothing, I don't see how a human being could sit and watch that and not do something. I left early for a reason, I think God had a lot to do with it. I was basically going to spectate, It's just adrenaline. You can't even imagine, I thought I could get in and get them out in time, That was my rationale: at least try. I thought, 'There's no way I'm going to get him out. He's going to die.' Three more minutes and I don't know if I would have made it out, You don't think about anything while it's happening.

	1 (1)	2 (2)	3 (3)	4 (4)	5 (5)	6 (6)	7 (7)
Intuitive/Fast:Reasoned/Slow (1)	❍	❍	❍	❍	❍	❍	❍

carn11 I was scared as soon as I got out there and realized that they couldn't pull us in, we both realized we could die. I thought about that I needed to focus and not think about dying,

	1 (1)	2 (2)	3 (3)	4 (4)	5 (5)	6 (6)	7 (7)
Intuitive/Fast:Reasoned/Slow (1)	❍	❍	❍	❍	❍	❍	❍

carn12 Oh, God yes, I didn't really think about it. I just jumped over (the dog).

	1 (1)	2 (2)	3 (3)	4 (4)	5 (5)	6 (6)	7 (7)
Intuitive/Fast:Reasoned/Slow (1)	❍	❍	❍	❍	❍	❍	❍

carn13 The thing I remember the most was that my adrenaline was pumping so hard that I didn't think that I would not get out of it, I never had the first qualm that we would not make it.

	1 (1)	2 (2)	3 (3)	4 (4)	5 (5)	6 (6)	7 (7)
Intuitive/Fast:Reasoned/Slow (1)	❍	❍	❍	❍	❍	❍	❍

carn14 When I heard the screaming, all I thought was I could not let someone burn to death, because I knew somebody in there did not have much time.If he had just called 911, there is no way that girl would have survived because that car was totally engulfed in flames one minute after I pulled her out, I just couldn't, in good conscience, do nothing.

	1 (1)	2 (2)	3 (3)	4 (4)	5 (5)	6 (6)	7 (7)
Intuitive/Fast:Reasoned/Slow (1)	❍	❍	❍	❍	❍	❍	❍

carn15 I just could not ignore it. Oh man, my heart was racing. It happened really quick. When you are looking at that gun, you are thinking, 'I could get shot here.' All I wanted to do was take his mind off that gun.

	1 (1)	2 (2)	3 (3)	4 (4)	5 (5)	6 (6)	7 (7)
Intuitive/Fast:Reasoned/Slow (1)	❍	❍	❍	❍	❍	❍	❍

carn16 I knew if we didn't get her out she would be overcome by smoke.

	1 (1)	2 (2)	3 (3)	4 (4)	5 (5)	6 (6)	7 (7)
Intuitive/Fast:Reasoned/Slow (1)	❍	❍	❍	❍	❍	❍	❍

carn17 I was thinking, ‘Maybe I should just shoot this guy.' But I knew I didn't want to end my life knowing I had killed another person, so I threw it way across the floor,

	1 (1)	2 (2)	3 (3)	4 (4)	5 (5)	6 (6)	7 (7)
Intuitive/Fast:Reasoned/Slow (1)	❍	❍	❍	❍	❍	❍	❍

carn18 I just did what I felt like I needed to do. You don't think about someone making that big a deal out of it.

	1 (1)	2 (2)	3 (3)	4 (4)	5 (5)	6 (6)	7 (7)
Intuitive/Fast:Reasoned/Slow (1)	❍	❍	❍	❍	❍	❍	❍

carn19 It was just the response I had. There was no time to call anybody. I had no choice, there was no one around.

	1 (1)	2 (2)	3 (3)	4 (4)	5 (5)	6 (6)	7 (7)
Intuitive/Fast:Reasoned/Slow (1)	❍	❍	❍	❍	❍	❍	❍

carn20 It's just I would hope everybody would do that and it's just that I was the first one there. I know I was trying to move as fast as I could because I could feel the heat and see the flames from the front seat .

	1 (1)	2 (2)	3 (3)	4 (4)	5 (5)	6 (6)	7 (7)
Intuitive/Fast:Reasoned/Slow (1)	❍	❍	❍	❍	❍	❍	❍

carn21 my first thought [was] maybe I can help, because I'm more used to dealing with traumatic situations like that. I have a clear head and a calm head to try to help those people, and luckily I did,

	1 (1)	2 (2)	3 (3)	4 (4)	5 (5)	6 (6)	7 (7)
Intuitive/Fast:Reasoned/Slow (1)	❍	❍	❍	❍	❍	❍	❍

carn22 I did anything and everything to get it off my dad ‘cause that’s my dad I was just at the right place at the right time.

	1 (1)	2 (2)	3 (3)	4 (4)	5 (5)	6 (6)	7 (7)
Intuitive/Fast:Reasoned/Slow (1)	❍	❍	❍	❍	❍	❍	❍

carn23 That's what drove me to do this. I didn't want another family going through the pain, “If [Adrian] can smile today, my daughter’s smiling too.

	1 (1)	2 (2)	3 (3)	4 (4)	5 (5)	6 (6)	7 (7)
Intuitive/Fast:Reasoned/Slow (1)	❍	❍	❍	❍	❍	❍	❍

carn24 I have a background in swimming in high school and all and I'm probably a little more comfortable than other people so it's the timing and the luck of it

	1 (1)	2 (2)	3 (3)	4 (4)	5 (5)	6 (6)	7 (7)
Intuitive/Fast:Reasoned/Slow (1)	❍	❍	❍	❍	❍	❍	❍

carn25 I myself would not be able to handle losing a kid, so for me to even think I could stand there and watch another mother lose her kid ... that’s the most horrible thought in the world, [My son] was on a life-support machine; we almost lost him,“I’ve had that nervous breakdown ... so I think that was part of my instinct. I’m a nobody, but I stopped and helped that kid because I’m a human being.

	1 (1)	2 (2)	3 (3)	4 (4)	5 (5)	6 (6)	7 (7)
Intuitive/Fast:Reasoned/Slow (1)	❍	❍	❍	❍	❍	❍	❍

carn26 I just saw it happen and decided to go after it, When I was younger I always wanted to have cool experiences and I kind of trained myself to be ready for acting in the moment.

	1 (1)	2 (2)	3 (3)	4 (4)	5 (5)	6 (6)	7 (7)
Intuitive/Fast:Reasoned/Slow (1)	❍	❍	❍	❍	❍	❍	❍

carn27 At first it is instinctive, You hear someone yell for help I tell my partner, 'We have to do something, or this lady is going to die.'

	1 (1)	2 (2)	3 (3)	4 (4)	5 (5)	6 (6)	7 (7)
Intuitive/Fast:Reasoned/Slow (1)	❍	❍	❍	❍	❍	❍	❍

carn28 It happened so fast. She encouraged me to go, in the heat of the moment having that confidence, trust and encouragement was really helpful, My memory of what I was thinking is that there were kids or other people in the car, that was my biggest fear. I remember not panicking, but the whole moment was pretty surreal, There was a brief moment where I thought about what the correct amount of clothing I should be wearing.

	1 (1)	2 (2)	3 (3)	4 (4)	5 (5)	6 (6)	7 (7)
Intuitive/Fast:Reasoned/Slow (1)	❍	❍	❍	❍	❍	❍	❍

carn29 Ethnicity doesn’t mean anything. I knew another human being was on the other side of that wall, and I knew I could never live with myself if I didn’t do something.

	1 (1)	2 (2)	3 (3)	4 (4)	5 (5)	6 (6)	7 (7)
Intuitive/Fast:Reasoned/Slow (1)	❍	❍	❍	❍	❍	❍	❍

carn30 Anybody would have done the same thing, I couldn’t leave them yelling for help. I had to do something. When I got out there I thought ‘OK big shot. What are you going to do now?’ I don’t know where I got the strength. I somehow changed from 75 to a young fella at that point.

	1 (1)	2 (2)	3 (3)	4 (4)	5 (5)	6 (6)	7 (7)
Intuitive/Fast:Reasoned/Slow (1)	❍	❍	❍	❍	❍	❍	❍

carn31 I just did what I had to do.

	1 (1)	2 (2)	3 (3)	4 (4)	5 (5)	6 (6)	7 (7)
Intuitive/Fast:Reasoned/Slow (1)	❍	❍	❍	❍	❍	❍	❍

carn32 I wasn't really thinking. It's scary, I was thinking that. I was there and I knew the fire department was coming and I knew Paul was coming and that he needed to be brought out of the building. ~mostly with my job the times where you don't have time to think, you're not nervous. There's not really a time to think about nervousness or being scared at that point. You're going to react one way or another.

	1 (1)	2 (2)	3 (3)	4 (4)	5 (5)	6 (6)	7 (7)
Intuitive/Fast:Reasoned/Slow (1)	❍	❍	❍	❍	❍	❍	❍

carn33 To be honest, I thought twice about jumping in because I know how cold that water is, But I had recently done a triathlon and I knew I had the training to do it. I knew by the time anyone else got to them, it would be too late.

	1 (1)	2 (2)	3 (3)	4 (4)	5 (5)	6 (6)	7 (7)
Intuitive/Fast:Reasoned/Slow (1)	❍	❍	❍	❍	❍	❍	❍

carn34 I already had my mind made up. As soon as it happened, there was only one outcome - to get him out, period, At the time, my one goal was to get him out of the car.

	1 (1)	2 (2)	3 (3)	4 (4)	5 (5)	6 (6)	7 (7)
Intuitive/Fast:Reasoned/Slow (1)	❍	❍	❍	❍	❍	❍	❍

carn35 When there wasn't screaming any more, it was life or death. There was no more thinking. I did it because I felt I needed to do it,

	1 (1)	2 (2)	3 (3)	4 (4)	5 (5)	6 (6)	7 (7)
Intuitive/Fast:Reasoned/Slow (1)	❍	❍	❍	❍	❍	❍	❍

carn36 He knew and I knew that if she saw that boat go away without her, she wouldn't be OK

	1 (1)	2 (2)	3 (3)	4 (4)	5 (5)	6 (6)	7 (7)
Intuitive/Fast:Reasoned/Slow (1)	❍	❍	❍	❍	❍	❍	❍

carn37 I had only two thoughts: one, I have to get him out of the door, and two, oh my God, this guy could kill me, too, I ended up on my back with the knife in my ribs, I think it was just instinct. Kind of like my tendency, that nobody in my platoon is going to get attacked without me doing something, if it were my daughter, you'd do it for me. You'd do it in an instant. And I'd do it for you.

	1 (1)	2 (2)	3 (3)	4 (4)	5 (5)	6 (6)	7 (7)
Intuitive/Fast:Reasoned/Slow (1)	❍	❍	❍	❍	❍	❍	❍

carn38 I thought about that before the cage came the first time. Before the cage came I said my prize (?) because once I saw that thing bobbing and I thought it was a machine I thought about them kids and the wife and I said 'That's it' You know, its over. It's like a glass, there's one way in and one way out and where we got to go out that's where all the water's coming from and you where on the ground I figured the whole thing was going to collapse but when I went up the first time I didn't think twice before I know I made it out I gotta go back and get them. It's very difficult to work with a guy everyday and walk away and leave him you can't do it. You know you gotta get him A lot of the times it's not the same decision to make like for instance they didn't have any time.

	1 (1)	2 (2)	3 (3)	4 (4)	5 (5)	6 (6)	7 (7)
Intuitive/Fast:Reasoned/Slow (1)	❍	❍	❍	❍	❍	❍	❍

carn39 In retrospect, it was the only choice we could make, You could either choose to watch someone die or react. [I] did what I needed to do.

	1 (1)	2 (2)	3 (3)	4 (4)	5 (5)	6 (6)	7 (7)
Intuitive/Fast:Reasoned/Slow (1)	❍	❍	❍	❍	❍	❍	❍

carn40 My plan was to a) not die trying to rescue this guy and b) try to rescue this guy. and that's about all I really had as a plan. There was an instinctive moment I wanted to clock the guy and take his rope from him and try to save the guy and this was all in like a milisecond because my adrenaline is running and I'm in overdrive and I mean it must have been one split second later that he just went floating right on by them and they probably felt pretty stupid. I took off like a jackrabbit as fast as I could.

	1 (1)	2 (2)	3 (3)	4 (4)	5 (5)	6 (6)	7 (7)
Intuitive/Fast:Reasoned/Slow (1)	❍	❍	❍	❍	❍	❍	❍

carn41 We thought about calling the Coast Guard on our cell phone, but I thought they wouldn’t get there in time,I remember James [the guide] saying to me, ‘Your safety is my priority.’ Then I said, ‘Well then his safety is my No. 1 priority.’

	1 (1)	2 (2)	3 (3)	4 (4)	5 (5)	6 (6)	7 (7)
Intuitive/Fast:Reasoned/Slow (1)	❍	❍	❍	❍	❍	❍	❍

carn42 I’ve got to get in the water. At that point, I acted immediately. I didn't think about it. I just started doing it.

	1 (1)	2 (2)	3 (3)	4 (4)	5 (5)	6 (6)	7 (7)
Intuitive/Fast:Reasoned/Slow (1)	❍	❍	❍	❍	❍	❍	❍

carn43 I just went for it, It was easy to get out to him. I was just doing what I hope someone would do for me, if I was there. It's just kind of common sense, I guess I really didn’t think about it too much.

	1 (1)	2 (2)	3 (3)	4 (4)	5 (5)	6 (6)	7 (7)
Intuitive/Fast:Reasoned/Slow (1)	❍	❍	❍	❍	❍	❍	❍

carn44 I wasn’t going to let this 12-year-old go under, I was determined to get her back to her parents.

	1 (1)	2 (2)	3 (3)	4 (4)	5 (5)	6 (6)	7 (7)
Intuitive/Fast:Reasoned/Slow (1)	❍	❍	❍	❍	❍	❍	❍

carn45 I just acted on instinct.

	1 (1)	2 (2)	3 (3)	4 (4)	5 (5)	6 (6)	7 (7)
Intuitive/Fast:Reasoned/Slow (1)	❍	❍	❍	❍	❍	❍	❍

carn46 It's a great honor, I'm proud that I was with my two friends and they did just as much as I did. From the very beginning, we really didn't think it was a big deal, I can't believe this is still going on and we're still being recognized. We didn't think about the magnitude of what we had done that day, until more people found out and all of the adults said how dangerous it was.

	1 (1)	2 (2)	3 (3)	4 (4)	5 (5)	6 (6)	7 (7)
Intuitive/Fast:Reasoned/Slow (1)	❍	❍	❍	❍	❍	❍	❍

carn47 It was one of those situations where, if it was a relative of mine or a friend of mine, I would want someone to do all that they thought they could do to help them.

	1 (1)	2 (2)	3 (3)	4 (4)	5 (5)	6 (6)	7 (7)
Intuitive/Fast:Reasoned/Slow (1)	❍	❍	❍	❍	❍	❍	❍

carn48 At that point, it was obvious to me, I got to act and work on rescuing them right there.

	1 (1)	2 (2)	3 (3)	4 (4)	5 (5)	6 (6)	7 (7)
Intuitive/Fast:Reasoned/Slow (1)	❍	❍	❍	❍	❍	❍	❍

carn49 I just realized she was in imminent danger, She survived the first fall, but I realized there was no way she would survive the second fall, which was a vertical cliff of about 40 feet. I was just in the right place at the right time, I thought, 'I have a son the same age,' and I figured this is what I would want someone to do if my son had fallen down, My first worry was that she would go into shock or a coma, My second concern was that I would slip off the cliff myself. I was surprised I was able to think about my first-aid training and utilize everything I've learned,

	1 (1)	2 (2)	3 (3)	4 (4)	5 (5)	6 (6)	7 (7)
Intuitive/Fast:Reasoned/Slow (1)	❍	❍	❍	❍	❍	❍	❍

carn50 From the very beginning, we really didn't think it was a big deal. We didn't think about the magnitude of what we had done that day,

	1 (1)	2 (2)	3 (3)	4 (4)	5 (5)	6 (6)	7 (7)
Intuitive/Fast:Reasoned/Slow (1)	❍	❍	❍	❍	❍	❍	❍

carn51 A higher power was there and watching over me and Mr. Perkins that night, There was definitely a higher power looking out for me and Mr. Perkins that night.

	1 (1)	2 (2)	3 (3)	4 (4)	5 (5)	6 (6)	7 (7)
Intuitive/Fast:Reasoned/Slow (1)	❍	❍	❍	❍	❍	❍	❍

INTUITIVE CONTROLS

intu_con1 I'm a media major, and have recently become curious about sociology. So I registered for a class at the local Community College. Within 10 minutes the teacher clearly hated me. I tend to stick things out, and I rarely back down from confrontation. But my instinct was to drop the class. After consideration . the teacher was hostile, the class novice, and the course based strictly on regurgitating the text rather than research papers . I did drop it. No pain, no aggravation, and I can read the text on my own.

	1 (1)	2 (2)	3 (3)	4 (4)	5 (5)	6 (6)	7 (7)
Intuitive/Fast:Reasoned/Slow (1)	❍	❍	❍	❍	❍	❍	❍

intu_con2 My daughter attended day care. Though she was doing fine and seemed to be having fun, I had a gut feeling something was wrong. I came to pick her up early one day to find that no teacher were in the room and five toddlers were running about.

	1 (1)	2 (2)	3 (3)	4 (4)	5 (5)	6 (6)	7 (7)
Intuitive/Fast:Reasoned/Slow (1)	❍	❍	❍	❍	❍	❍	❍

intu_con3 there are few times when my intuition led me in right direction.one of them is my decision which i took between working / studying after completing my graduation . my intuition said go for the post graduation even when i had a job offer in the hand, well now i am a masters degree holders and have a better job.

	1 (1)	2 (2)	3 (3)	4 (4)	5 (5)	6 (6)	7 (7)
Intuitive/Fast:Reasoned/Slow (1)	❍	❍	❍	❍	❍	❍	❍

intu_con4 When I chose a game in casino, i won a lot of money

	1 (1)	2 (2)	3 (3)	4 (4)	5 (5)	6 (6)	7 (7)
Intuitive/Fast:Reasoned/Slow (1)	❍	❍	❍	❍	❍	❍	❍

intu_con5 When i play video games. Its like i know that a guy will be in certain places. And I anticipate things all the time. I know exactly where to throw grenades. It is crazy that it happens so often. Like it happens every day. My intuition is strong.

	1 (1)	2 (2)	3 (3)	4 (4)	5 (5)	6 (6)	7 (7)
Intuitive/Fast:Reasoned/Slow (1)	❍	❍	❍	❍	❍	❍	❍

intu_con6 My daughter was ill with a cold. She did not seem to be getting better. I felt that she might have pneumonia. I took her to the doctor and she did. She was treated promptly and made a complete recovery quickly

	1 (1)	2 (2)	3 (3)	4 (4)	5 (5)	6 (6)	7 (7)
Intuitive/Fast:Reasoned/Slow (1)	❍	❍	❍	❍	❍	❍	❍

intu_con7 During my sophomore year, I had been offered a position at the administration building. My resume was due to my instructor about an hour after I had been informed of the position, but I took the leap and signed up for an interview. My first instinct led me in the right direction in this case because I was the only applicant for the job and I ended up working at the office for two years.

	1 (1)	2 (2)	3 (3)	4 (4)	5 (5)	6 (6)	7 (7)
Intuitive/Fast:Reasoned/Slow (1)	❍	❍	❍	❍	❍	❍	❍

intu_con8 back when everybody was buying dot.com stocks in early 2000, I had a feeling about gold. So I went with my gut. While evryone else watched their stuff lose all it's value, I am sitting pretty because of my 'gut'.

	1 (1)	2 (2)	3 (3)	4 (4)	5 (5)	6 (6)	7 (7)
Intuitive/Fast:Reasoned/Slow (1)	❍	❍	❍	❍	❍	❍	❍

intu_con9 I was accepted to 4 colleges with equivalent programs and tuition costs. I chose the school that seemed to provide the best fit . based on instinct. This included the type of buildings. This also included the type of people on campus. I toured all 4 schools and knew, by instinct, which school was best. I graduated and enjoyed my time at school. It was a good decision.

	1 (1)	2 (2)	3 (3)	4 (4)	5 (5)	6 (6)	7 (7)
Intuitive/Fast:Reasoned/Slow (1)	❍	❍	❍	❍	❍	❍	❍

intu_con10 My husband and I were invited to a party, but I really felt like we shouldn't go, and that we would have more fun at home. As it turns out, very few people attended the party, and a couple of friends ended up getting in a fight. We avoided a tense, awkward and boring evening.

	1 (1)	2 (2)	3 (3)	4 (4)	5 (5)	6 (6)	7 (7)
Intuitive/Fast:Reasoned/Slow (1)	❍	❍	❍	❍	❍	❍	❍

intu_con11 I once had to choose between two jobs that looked equally good on paper, but one just felt like it was the right fit for me. It turned out to be the best job I've ever had.

	1 (1)	2 (2)	3 (3)	4 (4)	5 (5)	6 (6)	7 (7)
Intuitive/Fast:Reasoned/Slow (1)	❍	❍	❍	❍	❍	❍	❍

intu_con12 In my family crisis my instinct told me to tell everything to my children.When I told them they understood everything and they guide me to take good decision and I now overcome the problem.Even now I didn't tell it to my parants.

	1 (1)	2 (2)	3 (3)	4 (4)	5 (5)	6 (6)	7 (7)
Intuitive/Fast:Reasoned/Slow (1)	❍	❍	❍	❍	❍	❍	❍

intu_con13 My first instinct 7 years ago was to chat online with a girl with who I had never chatted with before. This was a very good move because 7 years later that girl is my wife and I am so very much in love with her to this day.

	1 (1)	2 (2)	3 (3)	4 (4)	5 (5)	6 (6)	7 (7)
Intuitive/Fast:Reasoned/Slow (1)	❍	❍	❍	❍	❍	❍	❍

intu_con14 I go to college daily. but i don't want to go to tuition for study because it's very hateful job you know. My first instinct when one person slapped me after that i saw the right direction that continuous still yet. Also i have a strong instinct sense of humor that different than all people. I proud that god gives me that. Thanks god.

	1 (1)	2 (2)	3 (3)	4 (4)	5 (5)	6 (6)	7 (7)
Intuitive/Fast:Reasoned/Slow (1)	❍	❍	❍	❍	❍	❍	❍

intu_con15 I was selected for two jobs. i closed my eyes and the job of my preference came to my mind. I joined that job. After few weeks a good supervisory post got vacant and I got promoted immediately. Had I gone for the other job, I would have remained in the worker level.

	1 (1)	2 (2)	3 (3)	4 (4)	5 (5)	6 (6)	7 (7)
Intuitive/Fast:Reasoned/Slow (1)	❍	❍	❍	❍	❍	❍	❍

intu_con16 I was driving to my regular supermarket, and instinct told me to continue to the more expensive market further up the road. When I walked in I won a prize.

	1 (1)	2 (2)	3 (3)	4 (4)	5 (5)	6 (6)	7 (7)
Intuitive/Fast:Reasoned/Slow (1)	❍	❍	❍	❍	❍	❍	❍

intu_con17 I moved to a new city about a year ago. I was looking for a job on Craigslist and found a listing that looked interesting. I thought to myself, Why not? My first instinct was to apply for the job even though I wasn't sure if I would get it. I ended up getting hired and I have been working there for almost 10 months.

	1 (1)	2 (2)	3 (3)	4 (4)	5 (5)	6 (6)	7 (7)
Intuitive/Fast:Reasoned/Slow (1)	❍	❍	❍	❍	❍	❍	❍

intu_con18 On the 26th of July 2005, when I was in junior college, I decided to skip college that day since I had a feeling that something was amiss and I also felt I needed a day off. That very day it rained 944mm within a 12 hour span, and it was also the highest rainfall recorded in a 24hr span anywhere in the world. Everything was flooded, people were stranded and many lives were lost. I was very relieved that I decided to stay home that day.

	1 (1)	2 (2)	3 (3)	4 (4)	5 (5)	6 (6)	7 (7)
Intuitive/Fast:Reasoned/Slow (1)	❍	❍	❍	❍	❍	❍	❍

intu_con19 When I had to get married, my first instinct about my husband was that he was a very nice guy. Then I did go ahead and marry him. He did turn out to be a very nice person. So I trust my instincts about people.

	1 (1)	2 (2)	3 (3)	4 (4)	5 (5)	6 (6)	7 (7)
Intuitive/Fast:Reasoned/Slow (1)	❍	❍	❍	❍	❍	❍	❍

intu_con20 When i was younger i was walking home and my friend wanted me to take a short cut with her and i said no because it did not feel right to me. Later that day i found out that she had gotten lost and they final found her a she was grounder because she was not suppose to go that way.

	1 (1)	2 (2)	3 (3)	4 (4)	5 (5)	6 (6)	7 (7)
Intuitive/Fast:Reasoned/Slow (1)	❍	❍	❍	❍	❍	❍	❍

intu_con21 When i play video games. Its like i know that a guy will be in certain places. And I anticipate things all the time. I know exactly where to throw grenades. It is crazy that it happens so often. Like it happens every day. My intuition is strong.

	1 (1)	2 (2)	3 (3)	4 (4)	5 (5)	6 (6)	7 (7)
Intuitive/Fast:Reasoned/Slow (1)	❍	❍	❍	❍	❍	❍	❍

intu_con22 I recently left my job at an electronics company for a job in the cell phone industry. I had a feeling I would make more money, and I am now making double what I was making.

	1 (1)	2 (2)	3 (3)	4 (4)	5 (5)	6 (6)	7 (7)
Intuitive/Fast:Reasoned/Slow (1)	❍	❍	❍	❍	❍	❍	❍

intu_con23 recently, I urged my husband to buy a piece of property which he didn't like first. i felt that we could get a profit if we resell it. like i thought just after three months, we got a big profit out of it

	1 (1)	2 (2)	3 (3)	4 (4)	5 (5)	6 (6)	7 (7)
Intuitive/Fast:Reasoned/Slow (1)	❍	❍	❍	❍	❍	❍	❍

intu_con24 one day i wanted to travel for one function by bus but that day early morning my intutuion told me not to go and i acted to it accordingly and it saved my life that day that bus which i had to go met with a severe accident escaped it with the help of my intutuion.

	1 (1)	2 (2)	3 (3)	4 (4)	5 (5)	6 (6)	7 (7)
Intuitive/Fast:Reasoned/Slow (1)	❍	❍	❍	❍	❍	❍	❍

intu_con25 It was two years before in 2008. I was in search of a nice mobile phone, shopkeeper showed me a Chinese model, it looked great but it was without any guarantee. I was in love with that set on first site. I took that model its working alright till now.

	1 (1)	2 (2)	3 (3)	4 (4)	5 (5)	6 (6)	7 (7)
Intuitive/Fast:Reasoned/Slow (1)	❍	❍	❍	❍	❍	❍	❍

intu_con26 I have good people judgment. When I worked at a tutoring center, I was in charge of hiring. One person's resume looked good, and she interviewed well. Discussing the matter with other members of management, we thought she seemed appropriate for the job. However, my intuition thought she seemed like she might not be appropriate. We turned her down. Later, we found out that she had trouble at other jobs.

	1 (1)	2 (2)	3 (3)	4 (4)	5 (5)	6 (6)	7 (7)
Intuitive/Fast:Reasoned/Slow (1)	❍	❍	❍	❍	❍	❍	❍

REFLECTIVE CONTROLS

refl_con1 I was not sure who I should hire to install my air conditioning. I went and got three different quotes. I talked to a friend who works in the HVAC industry for advice. I decided which model to purchase. I decided which contractor to go with. I got a good deal, and a good installation.

	1 (1)	2 (2)	3 (3)	4 (4)	5 (5)	6 (6)	7 (7)
Intuitive/Fast:Reasoned/Slow (1)	❍	❍	❍	❍	❍	❍	❍

refl_con2 While feeding out the cattle this morning I had to get though a mob of hungry cows while opening, closing and driving through the gate. I thought about it and simply angled the gate into the mob and pushing through in the tractor.

	1 (1)	2 (2)	3 (3)	4 (4)	5 (5)	6 (6)	7 (7)
Intuitive/Fast:Reasoned/Slow (1)	❍	❍	❍	❍	❍	❍	❍

refl_con3 I had completed my Diploma in Electronics and Communications. As I am not from a rich family, my parents did not want me to continue with studies. But after a lot of reasoning, I decided that I would study Bachelor of engineering. And I can already see the positive outcome of my decision.

	1 (1)	2 (2)	3 (3)	4 (4)	5 (5)	6 (6)	7 (7)
Intuitive/Fast:Reasoned/Slow (1)	❍	❍	❍	❍	❍	❍	❍

refl_con4 I had to decide between keeping my current job or moving to a new one. I considered my current salary, which is good and the general job market, which is bad. I also considered my need for health benefits which is a major consideration, and my overall health. In the end I decided to stay at my current job. I may not be happy here, but I think for now staying is the right decision.

	1 (1)	2 (2)	3 (3)	4 (4)	5 (5)	6 (6)	7 (7)
Intuitive/Fast:Reasoned/Slow (1)	❍	❍	❍	❍	❍	❍	❍

refl_con5 I was trying to solve a computer problem at work. I searched online and found two different solutions. I tried both solutions and neither worked. After working through each solution I found that by combining the two I could solve the problem.

	1 (1)	2 (2)	3 (3)	4 (4)	5 (5)	6 (6)	7 (7)
Intuitive/Fast:Reasoned/Slow (1)	❍	❍	❍	❍	❍	❍	❍

refl_con6 I don't usually "reason through" situations. I am very impulsive. There are a couple times I can think of where I thought things through. Right after moving in with my boyfriend, we wanted to buy a kitten. I wasn't sure if we could afford it. We figured out about how much a month paying for a kitten would cost. Then we decided to try to set aside that money and make it part of our budget. We did so easily, so we decided to get a kitten. Now our kitten is a part of our family.

	1 (1)	2 (2)	3 (3)	4 (4)	5 (5)	6 (6)	7 (7)
Intuitive/Fast:Reasoned/Slow (1)	❍	❍	❍	❍	❍	❍	❍

refl_con7 I wanted to secure an increase to my salary. Knowing that my current employer was miserly but in need of my expertise, I leveraged them by applying and accepting another position for more salary. By having an offer in hand I had the ability to strong arm my current employer for additional income/benefits or just go to the new employer. Instead of quitting, I gave myself options by interviewing while employed.

	1 (1)	2 (2)	3 (3)	4 (4)	5 (5)	6 (6)	7 (7)
Intuitive/Fast:Reasoned/Slow (1)	❍	❍	❍	❍	❍	❍	❍

refl_con8 When we went to foreign country which has relatively high criminal rate we were warned about security precautions and we always consider this. When we were robbed we decided to listen to those people and react calm and leave all the things to robbers without fight. Later the police man told us that we were lucky because normaly thing like this ends in shooting.

	1 (1)	2 (2)	3 (3)	4 (4)	5 (5)	6 (6)	7 (7)
Intuitive/Fast:Reasoned/Slow (1)	❍	❍	❍	❍	❍	❍	❍

refl_con9 I always used to believe in Allopathy treatment.One fine day I became 90%deaf. The doctors told me That I would have to keep a hearing aid. Later my friend told me to change to homoeopathic treatment. I did a thorough research about homoepathic treatment. I took medications. After six months as if a miracle. I became completely alright.

	1 (1)	2 (2)	3 (3)	4 (4)	5 (5)	6 (6)	7 (7)
Intuitive/Fast:Reasoned/Slow (1)	❍	❍	❍	❍	❍	❍	❍

refl_con10 Upon experiencing an equipment failure, I stood back and reviewed all of the possible failure modes, then, step by step disassembled the equipment, eliminating possibilities, until the failed part was identified. Logical processes lead to conclusions whereas if multiple tasks are performed at the same time, the root cause failure may be overlooked, or misidentified.

	1 (1)	2 (2)	3 (3)	4 (4)	5 (5)	6 (6)	7 (7)
Intuitive/Fast:Reasoned/Slow (1)	❍	❍	❍	❍	❍	❍	❍

refl_con11 Some days back i searched in the net for a good online job and i found out some websites where we can able to earn money through online but i could able to find that only two or three websites legitimate so i again did a research about the sites which i found legitimate and then i started working on it and i started earning from it.

	1 (1)	2 (2)	3 (3)	4 (4)	5 (5)	6 (6)	7 (7)
Intuitive/Fast:Reasoned/Slow (1)	❍	❍	❍	❍	❍	❍	❍

refl_con12 There was this one time that careful reasoning led me to a right direction and resulted in a good outcome. It was when I decided to work for my company now. I made the right decision in coming back again in this company where i became regular. I really strive hard and do the best i can to get through the company which i successfully did.

	1 (1)	2 (2)	3 (3)	4 (4)	5 (5)	6 (6)	7 (7)
Intuitive/Fast:Reasoned/Slow (1)	❍	❍	❍	❍	❍	❍	❍

refl_con13 When I was studying for my M.B.A, we have decision making groups. I was the leader of that. And every time we are getting some subjects. We discussed on it. And finally we are taking a decision on it. Almost all time, I am the final decision maker. Many time I am awarded for that.

	1 (1)	2 (2)	3 (3)	4 (4)	5 (5)	6 (6)	7 (7)
Intuitive/Fast:Reasoned/Slow (1)	❍	❍	❍	❍	❍	❍	❍

refl_con14 Deciding about what college to take for my graduation was the decision which I made by lot of thinking and careful reasoning .I consulted all my elders and the people who might direct me to correct choice.I finally analysed and calculated through the reasoning process which lead me to the correct choice of college and of the field in which I was interested.

	1 (1)	2 (2)	3 (3)	4 (4)	5 (5)	6 (6)	7 (7)
Intuitive/Fast:Reasoned/Slow (1)	❍	❍	❍	❍	❍	❍	❍

refl_con15 I had to decide whether or not to marry my husband. I had been hurt several times in the past by making bad decisions. I had to carefully consider the personality, compatibility, character, and physical characteristics of my future husband in order to make a good decision. We have been happily married for 25 yrs. now. I think I made the right decision.

	1 (1)	2 (2)	3 (3)	4 (4)	5 (5)	6 (6)	7 (7)
Intuitive/Fast:Reasoned/Slow (1)	❍	❍	❍	❍	❍	❍	❍

refl_con16 My husband and I had a salesman over to build a cover over our patio, it was very expensive and high pressure, we excused ourselves and talked it over alone and determined it was not the right thing for us, it is very easy to get sucked into doing something that you are not 100 percent sure of.

	1 (1)	2 (2)	3 (3)	4 (4)	5 (5)	6 (6)	7 (7)
Intuitive/Fast:Reasoned/Slow (1)	❍	❍	❍	❍	❍	❍	❍

refl_con17 Once at work, quite surprisingly, most of the employees failed to turn out. WE pondered over the reason for the same. We then called up each of the employees and requested them to come and express their grievances. But they were ready to come only in a group which we denied. Then they came individually, expressed regret over the happenings and pledged support.

	1 (1)	2 (2)	3 (3)	4 (4)	5 (5)	6 (6)	7 (7)
Intuitive/Fast:Reasoned/Slow (1)	❍	❍	❍	❍	❍	❍	❍

refl_con18 when confronted at a bar by a drunk male, he initially pushed me but instead of lashing back out i reasoned with myself and him avoiding any fight spiraling out of control

	1 (1)	2 (2)	3 (3)	4 (4)	5 (5)	6 (6)	7 (7)
Intuitive/Fast:Reasoned/Slow (1)	❍	❍	❍	❍	❍	❍	❍

refl_con19 When the stock market crashed in 2008, I have huge amount of paper loss in stock. Instead of selling most of my stock and taking loss, I evaluate the market condition as well as health of the financial system, and bought quite a few of financial stock around the beginning of 2009. I have made good profit now from that buying decision and recover my loss too.

	1 (1)	2 (2)	3 (3)	4 (4)	5 (5)	6 (6)	7 (7)
Intuitive/Fast:Reasoned/Slow (1)	❍	❍	❍	❍	❍	❍	❍

refl_con20 I failed in my 1st year examinations but I was a bit confused because I believed I had studied well. So i sat and analyzed my problem and prepared carefully next time and this time i passed with high marks.

	1 (1)	2 (2)	3 (3)	4 (4)	5 (5)	6 (6)	7 (7)
Intuitive/Fast:Reasoned/Slow (1)	❍	❍	❍	❍	❍	❍	❍

refl_con21 One time I made a reasonable decision. I had to choose to either go home walking at night or ask for a ride. I thought about it for some time. If I walked home, it could be dangerous. If I asked for a ride, I don't know if I would be bothering the person. I thought about both choices. I decided to ask for a ride. It was the reasonable thing to do.

	1 (1)	2 (2)	3 (3)	4 (4)	5 (5)	6 (6)	7 (7)
Intuitive/Fast:Reasoned/Slow (1)	❍	❍	❍	❍	❍	❍	❍

refl_con22 In college, I had to decide what my career path would be. I liked writing. I did not have a great deal of passion for any specific area. I knew that I wanted a nice paycheck. After some study, I decided to become a lawyer. Since then, I have never regretted the decision.

	1 (1)	2 (2)	3 (3)	4 (4)	5 (5)	6 (6)	7 (7)
Intuitive/Fast:Reasoned/Slow (1)	❍	❍	❍	❍	❍	❍	❍

refl_con23 I was recently working on a spreadsheet which involved macros. Some of the macros wouldn't work because of a syntax problem, so I methodically went through and corrected the bad code.

	1 (1)	2 (2)	3 (3)	4 (4)	5 (5)	6 (6)	7 (7)
Intuitive/Fast:Reasoned/Slow (1)	❍	❍	❍	❍	❍	❍	❍

refl_con24 When looking for a new television I wasn't sure where to start. I spent alot of time researching online. I also spend time deciding what i needed based on my needs. After reading lots of consumer reports I finally found what I was looking for.

	1 (1)	2 (2)	3 (3)	4 (4)	5 (5)	6 (6)	7 (7)
Intuitive/Fast:Reasoned/Slow (1)	❍	❍	❍	❍	❍	❍	❍

refl_con25 I guess when we bought our last house. We didn't just pick the first house that we saw. Then we looked at many houses. After a while, we decided on which one we wanted. We decided on a price that we thought was way low, but due to some circumstances, we thought they may accept it. They did. So being patient got us a good price on a house.

	1 (1)	2 (2)	3 (3)	4 (4)	5 (5)	6 (6)	7 (7)
Intuitive/Fast:Reasoned/Slow (1)	❍	❍	❍	❍	❍	❍	❍

DEMOGRAPGHICS

gender Gender:

- Male (1)
- Female (2)

age Age:

ed Highest level of education completed:

- Less than a high school degree (1)
- High School Diploma (2)
- Vocational Training (3)
- Attended College (4)
- Bachelor’s Degree (5)
- Graduate Degree (6)
- Unknown (7)
